# Optimization method for human-robot command combinations of hexapod robot based on multi-objective constraints

**DOI:** 10.3389/fnbot.2024.1393738

**Published:** 2024-04-05

**Authors:** Xiaolei Chen, Bo You, Zheng Dong

**Affiliations:** ^1^The Key Laboratory of Intelligent Technology for Cutting and Manufacturing Ministry of Education, Harbin University of Science and Technology, Harbin, China; ^2^The Heilongjiang Provincial Key Laboratory of Complex Intelligent System and Integration, Harbin University of Science and Technology, Harbin, China

**Keywords:** hexapod robot, collaborative control, command combinations, driving fatigue, remote control

## Abstract

Due to the heavy burden on human drivers when remotely controlling hexapod robots in complex terrain environments, there is a critical need for robot intelligence to assist in generating control commands. Therefore, this study proposes a mapping process framework that generates a combination of human-robot commands based on decision target values, focusing on the task of robot intelligence assisting drivers in generating human-robot command combinations. Furthermore, human-robot state constraints are quantified as geometric constraints on robot motion and driver fatigue constraints. By optimizing and filtering the feasible set of human-robot commands based on human-robot state constraints, instruction combinations are formed and recommended to the driver in real-time, thereby enhancing the efficiency and safety of human-machine coordination. To validate the effectiveness of the proposed method, a remote human-robot collaborative driving control system based on wearable devices is designed and implemented. Experimental results demonstrate that drivers utilizing the human-robot command recommendation system exhibit significantly improved robot walking stability and reduced collision rates compared to individual driving.

## 1 Introduction

Different from conventional terrestrial moving equipment such as wheeled or tracked vehicle, legged robot’s track on ground is a series of discrete footprints, and this non-continuous support characteristic effectively increases its adaptability to the uneven road. Legged robots have fully studied from the structural characteristics and movement patterns of legged animals and insects. For example, quadruped robots have drawn inspiration from the musculoskeletal structures of animals like gazelles (Li et al., 2022a), cheetahs (Lei et al., 2022), and mice (Bing et al., 2023), as well as the movement patterns of quadruped animals (Massi et al., 2019). Considering the impressive load-bearing capacity and motion stability of arthropod leg structures, hexapod robots have also borrowed from creatures such as cockroaches (Massi et al., 2019), ants (Zhakypov et al., 2019), and lobsters (Shim et al., 2016). In recent years, an increasing number of scholars have fully recognized that hexapod robots, with non-continuous contact points with the ground, can adapt to terrain environments with geometric and physical feature variations. They exhibit high load-bearing capacity and stability, making them an ideal mobile system for outdoor environments.

Unlike conventional robots with simple structures, hexapod robots have as many as 18 degrees of freedom in their legs alone. This high level of complexity, especially when carrying out tasks in complex environments, can impose a heavy burden on the operator and significantly reduce the overall motion coordination of the robot. Therefore, conventional approaches to legged robot self-locomotion intelligence on uneven terrain have yielded increasingly complex self-training architectures. Many rely on training locomotion controller by reinforcement learning in simulation, then transplant the training result to real terrain. ETH Zurich’s ANYmal is one of the most promising legged systems of this kind (Wangbo et al., 2019). They deployed learning agile and dynamic motor skills for their quadrupedal robot system. Other systems use rapid adaptation training at the robot motors (Choi et al., 2023), which can be successful in 70% of the trials when walking downstairs along a hiking trail (Kumar et al., 2021).

However, research on autonomous intelligent systems for robots in recent years has shown that the emergence and development of artificial intelligence technology has provided many new methods for robot intelligence, greatly advancing the process of robot intelligence. As for autonomous intelligent systems for robots, it is a highly complex control system that integrates various functions such as environmental perception, dynamic decision-making and planning, behavior control, and execution. Due to the lack of human drivers’ ability to handle unexpected and imprecise events, the overall intelligence level, flexibility, and adaptability of the system have been greatly limited. This is particularly true for legged mobile robots, as their walking environments are mostly characterized by unknown and rugged complexity, making it difficult for them to rely solely on autonomous intelligent systems. In fact, legged mobile robots often use a human-in-the-loop collaboration approach to accomplish mobility tasks.

Different from early human-robot collaborative methods that required real-time switching of control between humans and robots (Merat et al., 2008, 2014; Eriksson and Stanton, 2016), the current mainstream human-robot collaboration method is human-in-the-loop coordination. According to the position of the human operator, it can mainly be divided into two categories: manned shared control and driver remote participation coordination. Among them, the first type, manned shared control, has been widely applied in the fields of intelligent manufacturing and intelligent driving of vehicles. For example, Ma proposed a shared steering controller based on Nash game strategy, considering the differences in human-machine goal consistency (Ma et al., 2019). They used a non-cooperative MPC method to model the interaction path tracking tasks between the driver and the automated system, achieving the correctness of cooperative path tracking control between the driver and the vehicle’s onboard intelligent system. Huang proposed a human-driver in-loop coordination/shared steering control framework, applying state space small gain theory to the driver-vehicle coupled system, enabling the onboard intelligent system to work in coordination with the driver to achieve ideal lane-keeping performance (Huang et al., 2019). In addition, manned shared control theory not only enables machine intelligence at the operational control layer (Zhou et al., 2022; Xu et al., 2023) but also starts to share human work at the motion planning layer of robots (Xu et al., 2023).

For the second type of human-in-the-loop collaborative method, namely driver remote participation coordination, it is mostly used for hexapod robots in underwater (Yoo et al., 2016; Picardi et al., 2020), planetary surface (Arm et al., 2023), resource extraction, and other hazardous environments. This is because the mobile operating environment poses risks that make it unsuitable for manned shared control of human-robots collaboration (Si et al., 2022). Li developed a new semi-autonomous bilateral control dual-master/single-slave tactile remote operation system for hexapod robots. Through this system, not only was the sharing of environmental haptic information between the robot and the operator achieved, but also the maneuverability and local autonomy of the robot’s remote operation system were improved (Li et al., 2022b). Schwarz developed a control system for the rescue robot Momaro that can perform multi-task collaborative processing (Schwarz et al., 2017). By coordinating multiple operators to manipulate the robot, they completed the supervision and control of the entire operation process of the robot. However, the main issue faced by driver remote participation coordination at present is that the status information between humans and robots cannot be timely exchanged, severely limiting the effectiveness of human-robots collaboration.

To address the issue of insufficient flow of status-constrained information between humans and machines, particularly the challenge of robots being unable to perceive drivers’ dynamically adjusting collaborative strategies, researchers utilize wearable physiological signal acquisition equipment to detect and assess driver states. For example, by wearing muscle electrical signal acquisition devices to sense and identify drivers’ motion intentions, facilitating interpersonal collaborative control (Zhang et al., 2022; Lyu et al., 2023). After obtaining driver status information, Seet determine the required assistance level based on the driver’s workload and performance, increasing the involvement of the assistance system when the driver is overloaded or distracted, and reducing the assistance level when the driver’s workload is moderate to ensure driving stability and safety (Seet et al., 2023). Nguyen proposed a human-machine collaborative steering control strategy considering driver behavior states (Nguyen et al., 2017). They allocate assistance weights based on the driver’s behavior state and use fuzzy control theory to address speed and assistance weight variability issues, reducing human-machine conflicts and enhancing collaborative performance between humans and vehicles. Bueno et al. analyzed the impact of changes in driver cognitive load on human-machine driving authority switching through simulating non-driving tasks, indicating that regardless of the cognitive load size, engaging in non-driving tasks negatively affects the switching of human-machine driving authority due to reduced concentration (Bueno et al., 2016). Additionally, in driver remote participation collaborative control, the intelligent system interacts with the driver using tactile, visual, and auditory information to stimulate driver focus, while Ji experimentally verified that using tactile seats effectively enhances driver focus during driving, thereby improving safety and smoothness during human-machine driving authority switching (Ji et al., 2011). Forster use voice prompts and warning sounds to alert drivers about upcoming authority switches (Forster and Naujoks, 2017). These methods aim to enhance mutual perception between humans and machines, utilizing perceptual information to promote and assist the emerging trend of remote collaborative control between drivers and robots more effectively.

Based on the above discussion, in this paper, we consider how to quantitatively analyze the state constraints between humans and robots in remote control mode, assisting drivers in forming reasonable human-robot collaborative control commands. Especially, we place great emphasis on the geometric motion constraints of hexapod robots in irregular terrains and the fatigue state constraints of drivers. Using these two types of human-robot constraint conditions, we filter the feasible set of all human-robot collaborative control command solutions. The selected human-robot commands combinations by the driver are then chosen and issued to the robot, greatly reducing the driver’s burden and enhancing the safety and efficiency of remote collaboration The remainder of this paper is divided into the following sections: Section 2 proposes the mapping process framework of human-robot decision target values to command combinations. Section 3 quantifies the geometric motion constraints of hexapod robots in irregular terrains and the fatigue state constraints of drivers. Experimental investigations are conducted in Section 4.

## 2 Method for generating command combinations from human-robot decision target values

### 2.1 Framework of overall process

For robots performing tasks in unstructured terrain environments, the complexity of behavioral decision-making and control by remote operators is a crucial issue that cannot be ignored. In particular, unlike structurally simple conventional wheeled robots, hexapod robots have as many as 18 degrees of freedom. If controlled one by one, it not only imposes a heavy driving burden on the driver but also significantly reduces the overall motion coordination of the robot. During the phase of issuing commands with high control workload, it is particularly necessary to utilize the intelligent system carried by the robot to assist in rapid and efficient command issuance, thereby reducing the workload of the driver.

Our team recorded and summarized the real-time decision-making and control processes of highly experienced hexapod robot drivers through a large number of experiments. After summarizing, it was found that both drivers and robot decision intelligence tend to focus on the top-level decision-making of hexapod robot motion behavior, specifically targeting the next moment’s target walking distance, walking speed, and walking direction of the hexapod robot, forming decision goal values mutually recognized by humans and machines. Furthermore, the driver or robot intelligence system then decomposes and maps the decision goal values into corresponding specific control commands. In this process, for the driver, instructions are formulated in the brain based on the observed environment and robot state information, as well as driving experience, and implemented through operating external hardware devices; for the robot, theoretical formulas are established based on the robot’s kinematic characteristics to autonomously calculate positions and speeds at the bottom execution layer and generate instructions.

**Figure 1:**
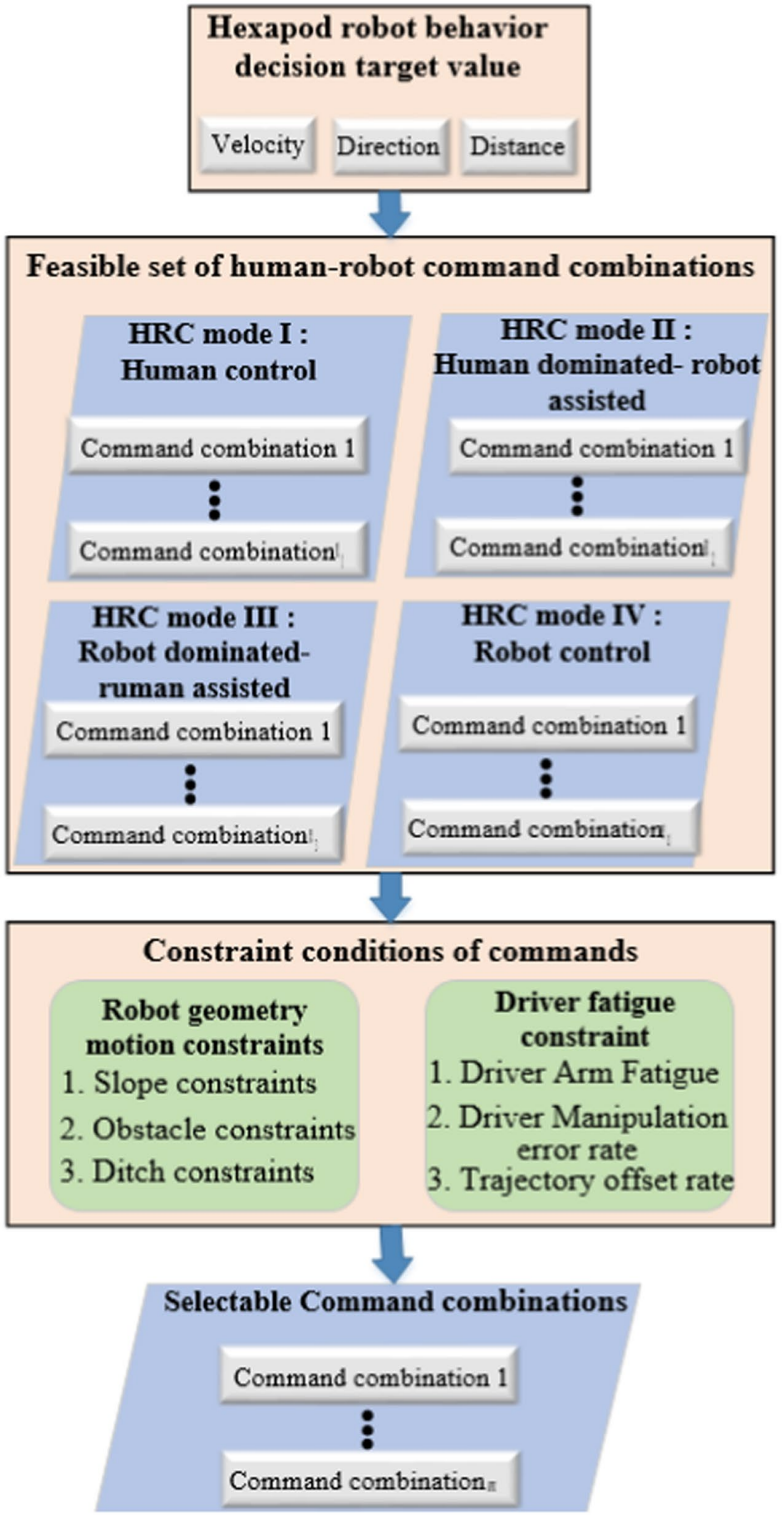
Mapping process framework of generating command combinations.

Specifically, as shown in Figure 1, this article outlines the main steps in the process from behavior decision goal values to recommended selectable human-robot command combinations as follows: (1) confirming and inputting behavior decision goal values; (2) mapping and calculating all human-robot commands from the decision goal values to form a feasible set of human-robot commands, including all four types of command combinations under human-robot collaborative modes (driver control, human primary and machine auxiliary, machine primary and human auxiliary, and robot autonomous mode); (3) filtering the command combinations in the feasible set based on command constraints, which include geometric motion constraints of the robot and driver fatigue constraints; (4) after filtering based on constraints, recommending human-robot commands are output to assist the driver in control.

For example, When a robot is moving in the slop terrain, a command combination generally includes the selection command for the robot’s gait type, commands for gait period, step stride, step stroke, and body posture adjustment. Moreover, the commands included in the combination correspond to specific recommended values and the authority for human-robot modifications. Therefore, the primary function of command combinations is to provide the human operator with the types, values, and permissions of recommended commands. Additionally, driver can make real-time modifications to the command online before the robot carries them out.

### 2.2 Hexapod robot motion characteristics

**Figure 2:**
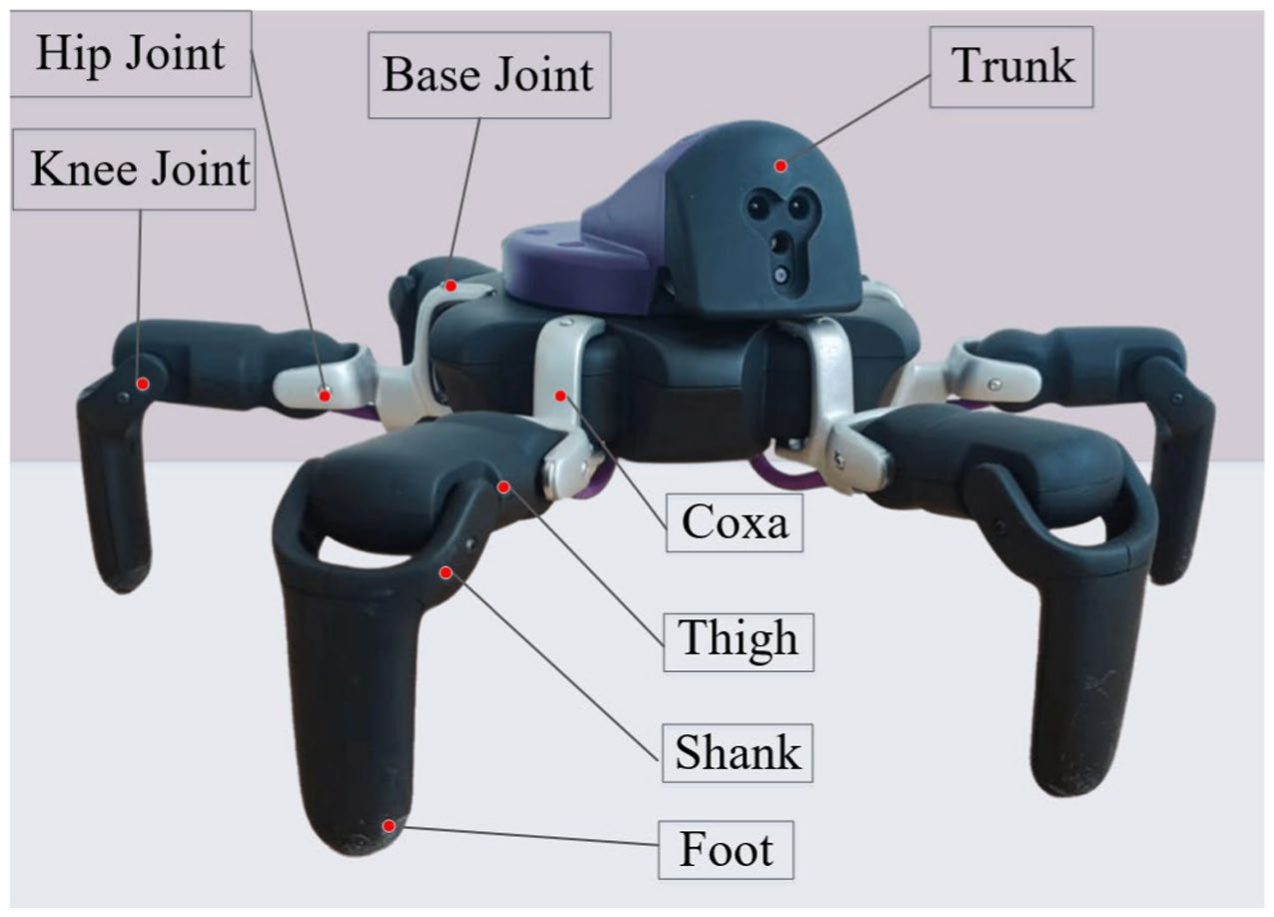
Hexapod robot physical prototype.

Unlike drivers who rely on experience to generate control commands, machine intelligence needs to establish a kinematic model based on robot motion characteristics to generate control commands. The physical prototype of the hexapod mobile robot is shown in Figure 2, which belongs to a type of insect-like electrically driven multi-legged robot. The robot mainly consists of a body and six legs. The body is hexagonal in shape, with the six legs evenly distributed on each side. Each leg has three degrees of freedom, composed of the coxa segment, thigh segment, and shank segment. The coxa segment is connected to the body via a base joint, the thigh segment is connected to the coxa segment by a hip joint, and the shank segment is connected to the thigh segment by knee joint. The robot’s foot is rigidly connected to the end of the shank segment. Each of the mentioned rotating joints is driven by a motor.

$\Sigma_{G-X_{1}Y_{1}Z_{1}}$ , $\Sigma_{B-X_{2}Y_{2}Z_{2}}$ , and $\Sigma_{L-X_{3}Y_{3}Z_{3}}$ represent the global, body and single-leg coordinate systems, respectively. The base joint angle is denoted by α , the hip joint angle is denoted by β , and the ankle joint angle is denoted by γ . The length of the coxa segment is represented by $L_{c}$ The length of the thigh segment is represented by $L_{t}$ , and the length of the is represented by $L_{s}$ . The vertical height from the body’s centroid to the ground is denoted by *H*. The forward kinematics and inverse kinematics models of a single leg of the hexapod mobile robot can be determined by Formula (1) and Formula (2):


(1) {PXL=Lc+Ltcosβ+Ls+LBcosγ+βsinαPYL=Lc+Ltcosβ+Ls+LBcosγ+βcosαPZL=−H+Ltsinβ+Ls+LBcosγ+β

(2) $$\left\{ \begin{array}{l} \alpha =\arctan\left(\frac{P0_{X}}{P0_{Y}}\right)\\ \begin{array}{c} \beta =\arctan\frac{P0_{Y}-L_{c}\cos\alpha }{\left(H-P0_{Z}\right)\cos\alpha }-\arcsin\left(\frac{\sqrt{{\left(H-P0_{Z}\right)}^{2}+{\left(\frac{PL_{Z}}{\cos\alpha }-L_{c}\right)}^{2}}}{2L_{t}}\right)\\ \;-\frac{{\left(L_{S}+L_{b}\right)}^{2}-{L^{2}}_{t}}{2L_{t}\sqrt{{\left(H-P0_{Z}\right)}^{2}+{\left(\frac{PL_{Z}}{\cos\alpha }-L_{c}\right)}^{2}}} \end{array}\\ \begin{array}{c} \gamma =\pi -\sin^{-1}\left(\begin{array}{l} \frac{\sqrt{{\left(H-PL_{z}\right)}^{2}+{\left(\frac{PL_{z}}{\cos\alpha }-L_{c}\right)}^{2}}}{2L_{s}}\\ +\frac{{L_{s}}^{2}-{L_{t}}^{2}}{2L_{s}\sqrt{{\left(H-PL_{z}\right)}^{2}+{\left(\frac{PL_{z}}{\cos\alpha }-L_{c}\right)}^{2}}} \end{array}\right)\\ \;-\sin^{-1}\left(\begin{array}{l} \frac{\sqrt{{\left(H-PL_{z}\right)}^{2}+{\left(\frac{PL_{z}}{\cos\alpha }-L_{c}\right)}^{2}}}{2L_{t}}\\ +\frac{{L_{s}}^{2}-{L_{t}}^{2}}{2L_{t}\sqrt{{\left(H-PL_{z}\right)}^{2}+{\left(\frac{PL_{z}}{\cos\alpha }-L_{c}\right)}^{2}}} \end{array}\right) \end{array} \end{array}\right.$$

## 3 Quantitative methods for human-robot state constraints

### 3.1 Geometric motion constraints of the robot

In order to ensure the safety of hexapod robots walking in complex terrain environments, it is necessary to impose specific constraints on the generated commands for both the robot and the driver based on terrain features. This article establishes geometric constraint models between terrain and joint space for sloped terrain, obstacle terrain, and ditch terrain, thereby ensuring that the robot’s joint motion space remains within a safe range. This includes constraint equations for joint motion based on terrain feature values, resulting in target step stride, step stroke, pitch, and roll angle constraints for the robot’s body pose changes.

Specifically, considering that terrain geometry features can greatly impact the robot’s joint motion space, the control process of the robot requires real-time monitoring of the joint’s safe working space to prevent issues such as joint position exceeding limits, instability and overturning during movement, and body collisions. In this section, we first utilize the robot’s body perception characteristics to establish terrain features, such as estimating the slope of sloped terrain, dimensions of obstacles in obstacle terrain, and the width of ditches in ditch terrain. Subsequently, based on constraints for robot body collision safety, joint limit constraints, and walking safety constraints, a mathematical model for the joint constraints of the hexapod robot is established. Finally, the constraints for the target commands for the robot’s body pose changes are obtained based on the constraints imposed by the terrain on the joints. This achieves the necessary rationalization of the feasible command set for human-robot instructions, narrowing the range of recommended commands while improving their rationality. This enhancement ensures that both the driver and the robot intelligence effectively improve the efficiency and safety of controlling the robot’s movement using the feasible command set.

#### 3.1.1 Geometric constraint model for sloped terrain

When a hexapod robot walks on sloped terrain, it needs to adjust the pitch and roll angles of its body as well as the step length in real time to adapt to the changing terrain based on the estimated slope of the ground and joint constraints. Specifically, when the robot is traversing sloped terrain, constraints need to be established based on the joint’s extreme positions or potential interference between the robot’s body and the geometric terrain, in order to obtain constraints for the numerical values of the hexapod robot’s motion commands.

**Figure 3:**
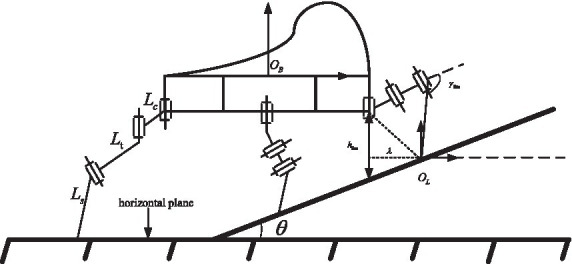
Robot in slope terrain.

Specifically, during the uphill process, in order to ensure the stability margin of the hexapod robot, a uniformly distributed standing method is adopted, as shown in Figure 3. When the terrain slope is steep, the knee joints of the front and rear legs will reach their limit positions. Therefore, by establishing geometric constraints on the height from the body to the slope surface, the geometric relationship between the terrain and the robot’s body can be mapped. The geometric relationships between the joints and the ground during the transition phase from flat ground to a slope for the hexapod robot are shown in Figure 3. The height of the front leg base joint position from the slope surface is determined by the knee joint’s limit position and the walking step length. The defined limit height of the base joint from the slope surface is denoted as $h_{\text{lim}}$ , with the vertical distance being the length of point AB. According to forward kinematic analysis, the limit position $\gamma_{\text{lim}}$ of the knee joint will mainly affect the value of $h_{\text{lim}}$ . Based on the limit height of $h_{\text{lim}}$ , the limit value of the knee joint position can be determined by the Formula (3):


(3) $$\begin{array}{l} \gamma_{\text{lim}}=\pi -\sin^{-1}\left(\begin{array}{l} \frac{\sqrt{{\left(h_{\text{lim}}-\lambda \tan\theta -PL_{z_{i}}\right)}^{2}+{\left(\frac{PL_{z_{i}}}{\cos\alpha }-L_{c}\right)}^{2}}}{2L_{s}}\\ +\frac{{L_{s}}^{2}-{L_{t}}^{2}}{2L_{s}\sqrt{{\left(h_{\text{lim}}-\lambda \tan\theta -PL_{z_{i}}\right)}^{2}+{\left(\frac{PL_{z_{i}}}{\cos\alpha }-L_{c}\right)}^{2}}} \end{array}\right)\\ -\sin^{-1}\left(\begin{array}{l} \frac{\sqrt{{\left(h_{\text{lim}}-\lambda \tan\theta -PL_{z_{i}}\right)}^{2}+{\left(\frac{PL_{z_{i}}}{\cos\alpha }-L_{c}\right)}^{2}}}{2L_{t}}\\ +\frac{{L_{s}}^{2}-{L_{t}}^{2}}{2L_{t}\sqrt{{\left(h_{\text{lim}}-\lambda \tan\theta -PL_{z_{i}}\right)}^{2}}+{\left(\frac{PL_{z_{i}}}{\cos\alpha }-L_{c}\right)}^{2}} \end{array}\right) \end{array}$$

(4) $$\lambda_{\text{max}}=\arg\max\;\left(\gamma \left(h_{\text{lim}},\lambda ,\theta \right)\right)$$

Since the limit position of the knee joint depends on the robot’s leg mechanical structure and joint motor limits, it is a fixed value. According to the Formula (3), it can be seen that the limit height of the base joint from the slope surface, the robot’s real-time step stride, and the slope angle will determine the real-time position of the knee joint. Considering that the limit height of the base joint from the slope surface is a predetermined value for safety reasons, and the slope angle is also an estimated determined value based on the robot’s body perception. Therefore, the real-time step length is an important factor determining the knee joint position in real time. To ensure that the knee joint’s limit position does not exceed its maximum set value, the real-time step length must not exceed a maximum limit value, as shown in Formula (4). By establishing the maximum real-time step length for a hexapod robot walking uphill, it can set practical constraints on step length. This will improve the effectiveness of instruction sets used by both the driver and the robot for controlling robot motion, enhancing human-robot interaction during driving.

#### 3.1.2 Geometric constraint model for obstacle terrain

**Figure 4:**
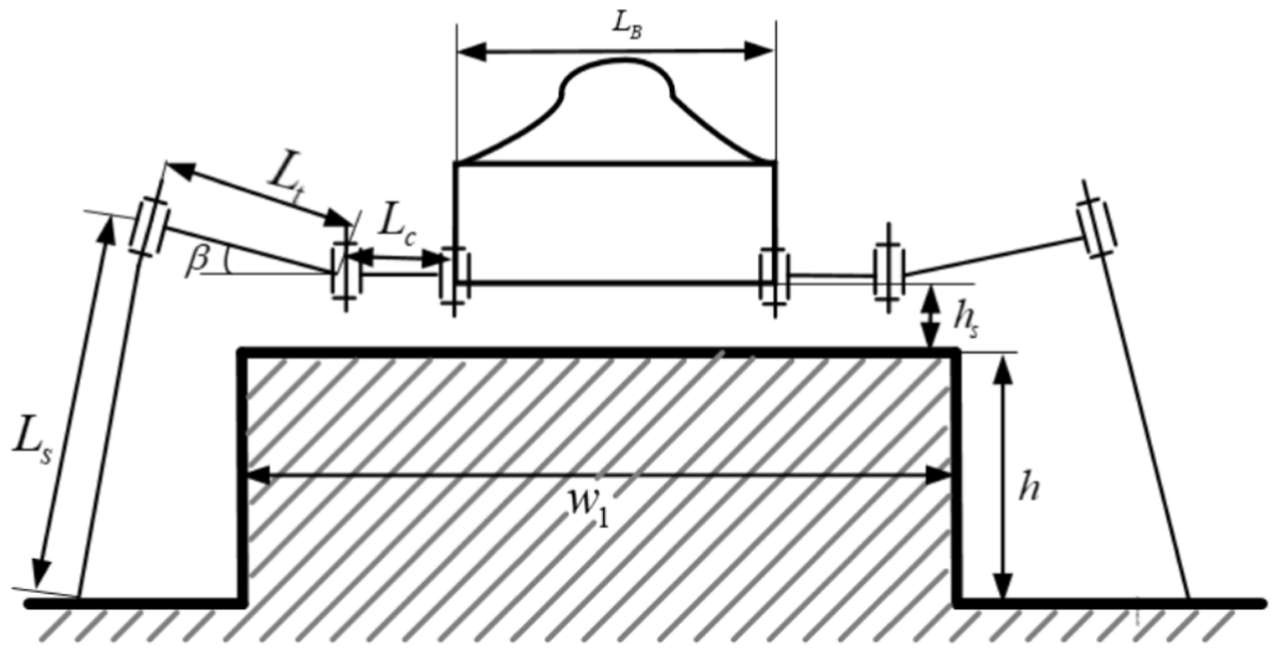
Obstacle that can be crossed.

**Figure 5:**
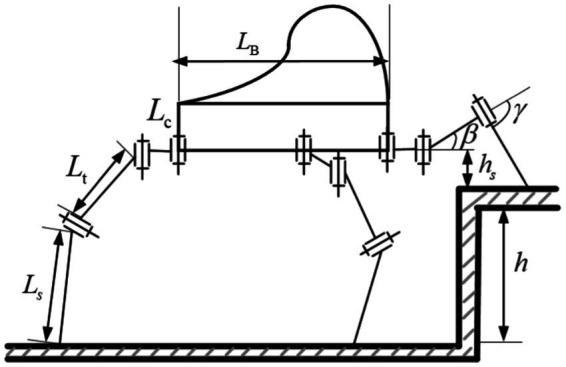
Obstacle that can be climbed.

Obstacle terrain is the most common non-flat terrain encountered by hexapod robots in complex outdoor environments. According to the geometric dimensions of the obstacles, obstacle terrain can be divided into two categories: obstacles that can be crossed (obstacle width is less than the leg support width, and obstacle height is lower than the robot’s standing height), as shown in Figure 4; obstacles that can be climbed (slope greater than the leg support width, and obstacle height is lower than the robot’s standing height), as shown in Figure 5. For obstacles that can be crossed, due to the long lengths of the robot’s thigh and shank joints, the robot’s standing height can be raised above the height of the obstacle terrain, and a normal walking gait can be used to pass through the obstacle terrain smoothly. For obstacles that can be crossed, when the robot’s standing height is greater than the height of the obstacle, the leg posture can be adjusted to achieve a new body standing height. The constraints that need to be satisfied in this state as shown in Formula (5).


(5) $$\left\{\begin{array}{l} L_{B}+2L_{c}+2L_{t}\cos\beta >w_{1}\\ L_{s}\sin\left(\gamma -\beta \right)-L_{s}\sin\beta >h+h_{s} \end{array}\right\}$$

Where $L_{c}$ represents the length of the hexapod robot’s leg base joint; $L_{t}$ represents the length of the hexapod robot’s leg tibia joint; $L_{s}$ represents the length of the hexapod robot’s leg femur joint; $L_{B}$ represents the width of the hexapod robot’s body, $w_{1}$ represents the width of a local obstacle in the terrain environment, h represents the height of a local obstacle in the terrain environment, and $h_{s}$ represents the safety distance between the hexapod robot’s leg base joint and the obstacle.

For obstacles in a climbable form, where the obstacle width is greater than the leg’s support width and the body height is lower than the maximum standing height, the legs can step on the obstacle and perform climbing actions. By setting a limit value $h_{s}$ for the distance between the leg base and the obstacle surface, we can determine the limit value $\eta_{\mathrm{robot}}$ for the body’s pitch angle and establish a geometric constraint model between joint space and obstacle terrain. When the robot’s front legs land on the obstacle surface, the joint motion space of the front legs is limited, requiring adjustment of the body’s pitch angle to adapt to the terrain changes. The constraints that need to be satisfied in this state as shown in Formula (6):


(6) $$\left\{\begin{array}{l} L_{t}\sin\beta +L_{s}\cos\;\left(\frac{\pi }{2}-\beta -\gamma \right)>h+h_{s}\\ \eta_{\mathrm{robot}}>\arccos\;\left(\frac{\sqrt{l^{2}-r^{2}}}{h_{s}}\right) \end{array}\right\}$$

Therefore, for climbable obstacles, the motion instructions of the hexapod robot adhere to the above constraints, effectively achieving reasonable and effective constraints on the pitch angle for both the driver and the robot intelligence when utilizing feasible instruction sets for robot motion control. This enhances the effectiveness of the feasible instruction set in assisting human-machine interaction during driving and control.

#### 3.1.3 Geometric constraint model for ditch terrain

**Figure 6:**
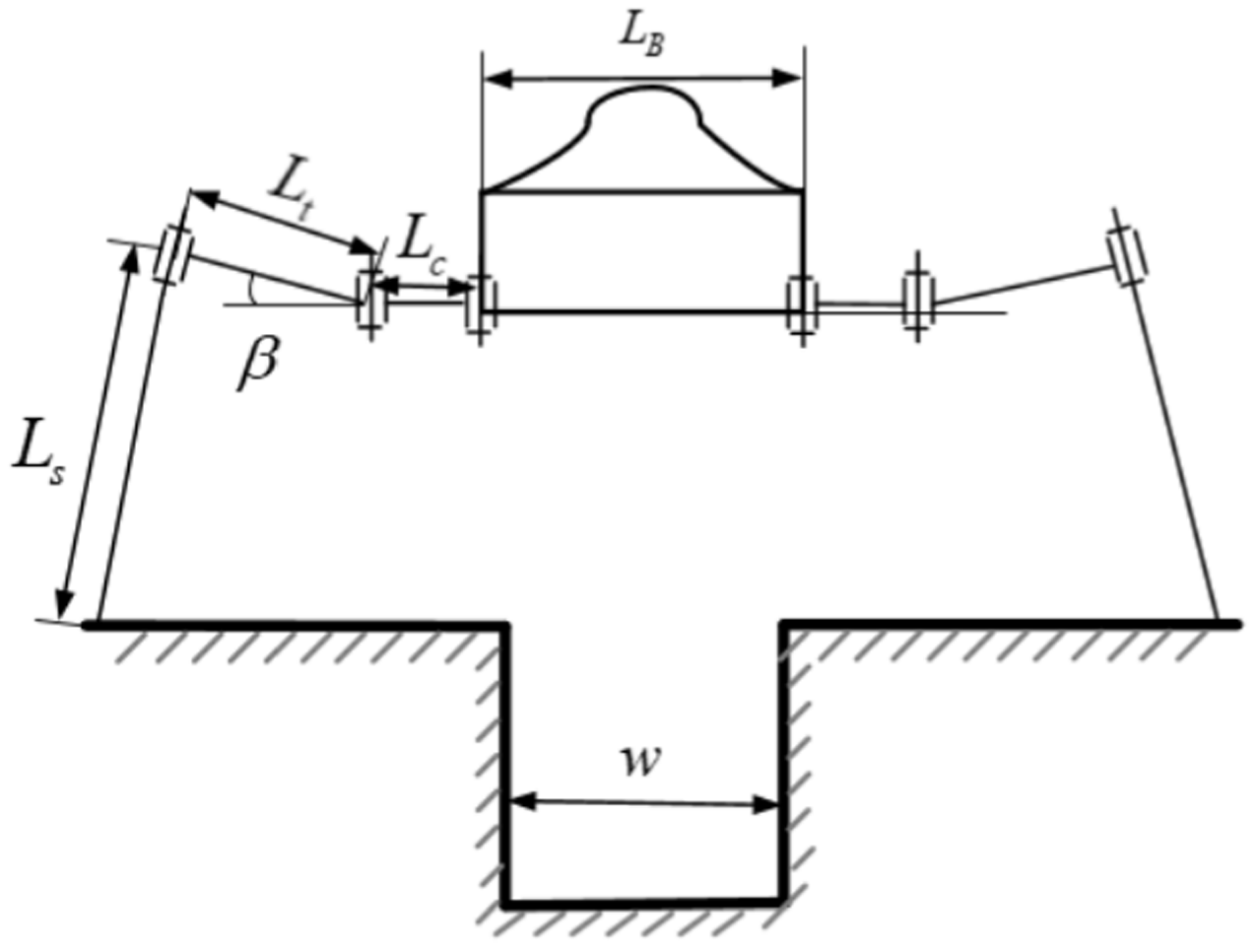
Ditch that can be crossed in a single step.

Based on the different geometric dimensions of the ditch terrain, the ditch terrain can be divided into two categories: ditches that can be crossed in a single step where the width of the channel is less than the robot’s single support width; and ridges that can be crossed in multiple steps where the width of the channel is greater than the robot’s single support width. For ridges that can be crossed in a single step, where the width of the channel is less than the robot’s single support width, the robot can increase its step length to autonomously cross the channel, as shown in Figure 6. The constraints that need to be satisfied in this state as shown in Formula (7):


(7) $$\lambda_{\text{min}}>w$$

Where $\lambda_{\text{min}}$ represents the real-time minimum step length of the hexapod robot, and w represents the width of the channel.

**Figure 7:**
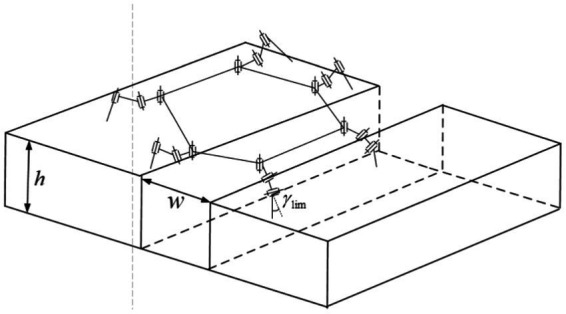
Ditch that can be crossed in multiple steps.

For ditches that can be crossed in multiple steps, where the width of the channel is greater than the robot’s single support width, it is not possible to cross the ridge with a single adjustment. However, the robot can achieve the crossing by making multiple adjustments with its legs. In this case, the supporting legs need to take larger steps, which may lead to situations where the joint reaches its limit position, as shown in Figure 7. The constraints that need to be satisfied in this state as shown in Formula (8):


(8) $$L_{s}+L_{t}\cos\beta -L_{c}\sin\;\left(\frac{\pi }{2}-\beta -\gamma_{\text{lim}}\right)-\lambda_{D}>w$$

Where $\gamma_{\text{lim}}$ represents the knee joint limit value, $\lambda_{D}$ represents the real-time dynamic step length of the hexapod robot, and w represents the width of the channel.

Through the above equation, the robot’s real-time dynamic maximum step stride can be calculated. When a single leg reaches its maximum step stride and cannot cross the channel, it is necessary to readjust the positions of each leg and the body, and then retry the crossing. Based on the geometric constraints model of the channel terrain mentioned above, autonomous step adjustment for the robot to cross the channel within the range of leg joint limit positions is achieved, enabling the robot to perform the crossing action. Moreover, when encountering obstacles that cannot be overcome or when the landing area is complex and requires finding a suitable landing point, external visual perception of the robot can be used to model the terrain and detect landing points. By modeling the terrain using external visual sensors and equivalent the robot’s envelope range to a virtual body model, obstacle detection and avoidance are carried out based on artificial potential field methods. Furthermore, analyzing the ruggedness of the terrain, terrain height, and the area of safe landing zones based on visual information, an evaluation function for the terrain is established to select landing points, avoiding instability of the robot caused by walking on special terrain.

Therefore, for ditches that can be crossed in multiple steps, the motion command s for hexapod robots should prioritize the constraints mentioned above. This will effectively realize the collaboration between the driver and the machine intelligence when using a feasible command set for robot motion control, providing reasonable and effective constraints on dynamic step length. It enhances the effectiveness of the feasible command set in assisting human-machine collaboration in driving and control tasks.

### 3.2 Driver fatigue constraint

Due to its inherent stability under high load and its ability to maneuver in extreme environments, hexapod robots are more likely to perform tasks in complex environments compared to other types of mobile robots. In order to ensure the passability and safety of hexapod robots in complex and unknown environments, remote operation and control of the robot’s motion behavior are often carried out through human-robot collaboration. However, the redundancy of the robot’s control degrees of freedom and the complexity of environmental tasks will impose a significant burden on the remote operators. This not only significantly affects the comfort of the operators but also has a detrimental impact on the safety and efficiency of the hexapod robot’s movement.

Therefore, it is necessary to assess the driver’s fatigue status in real-time to determine the optimal human-robot collaborative control mode, which can then be used to optimize the combined form of control commands for humans and robots. For example, when the driver is not fatigued or only mildly fatigued, the control system can switch to manual control mode, allowing the driver to participate in the position control of the hexapod robot’s single leg, foot end, and joints. When the driver is moderately fatigued, the control system can switch to human primary and machine auxiliary mode, enabling the driver to participate in the control of the hexapod robot’s body posture and gait parameters while disabling manual control mode. In cases of severe fatigue, the control system can switch to machine primary and human auxiliary mode, where the driver is only required to monitor and intervene in emergency situations concerning the hexapod robot.

**Figure 8:**
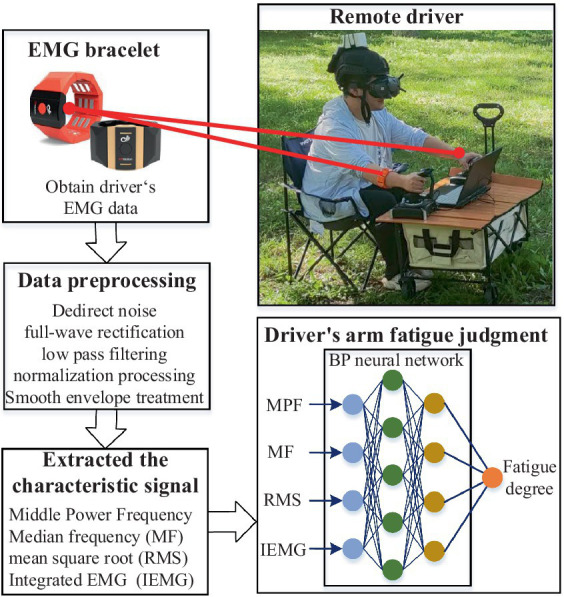
The remote human-machine collaborative driving control system.

As shown in Figure 8, for the quantitative analysis of driver arm fatigue, this paper designs a framework for quantifying upper limb fatigue. The main process includes: real-time collection of the driver’s raw electromyography signals from the upper limbs using a myoelectric armband; preprocessing the raw electromyography signals of the upper limbs using data processing methods to extract feature signals; training a BP neural network using the feature signals and the driver’s subjective fatigue values as training samples, thereby ultimately establishing and utilizing a neural network model for real-time assessment of driver arm fatigue.

Specifically, in the process of collecting the driver’s raw electromyography signals from the upper limbs, considering that electromyography signals are the electrophysiological signals generated when muscle tissue contracts, this paper collects 8-channel electromyography signal data using the gForcePro+ myoelectric acquisition armband. In the preprocessing stage of the raw signals, to improve the accuracy and anti-interference ability of the data, the sampling frequency of the signals is set to 1,000 Hz, and methods such as linear noise elimination, low-pass filtering, and moving average filtering are used to preprocess the original sEMG signals. This stage involves roughly five sub-processes: first, linear noise elimination for DC; second, square rectification of the obtained signals; third, further filtering of the rectified signals using filters; fourth, normalization of the processed signals; and fifth, moving average envelope processing of the normalized signals using a 50-sample moving window. The main formulas and their meanings involved in each process are as follows.

Sub-process 1: Denoising of the original signal by subtracting the mean amplitude of the signal from the signal amplitude within the window, as shown in Formula (9):


(9) $$S_{1}\left(i\right)=S_{0}\left(i\right)-\frac{1}{N}\overset{N-1}{\underset{i=0}{\sum }}S_{0}\left(i\right)$$

Where $S_{1}\left(i\right)$ represents the sEMG value with linear noise removed; $S_{0}\left(i\right)$ represents the value of the original sEMG signal; N represents the sampling window size; i represents the instantaneous moment of processing the sEMG signal.

Sub-process 2: Full-wave rectification of the signal obtained from process 1, as shown in the formula, as shown in Formula (10):


(10) $$S_{2}\left(i\right)=\mathrm{abs}\overset{N-1}{\underset{n=0}{\sum }}S_{1}\left(i\right)$$

Where $S_{2}\left(i\right)$ represents the amplitude of the sEMG signal after full-wave rectification, ensuring that the amplitude of the abs signal is entirely non-negative; N represents the sampling window size; i represents the instantaneous moment of processing the sEMG signal.

Sub-process 3: Using a 4th-order Butterworth bandpass filter to limit the frequency to the range of 30-100 Hz, the signal is processed to remove high-frequency noise through filtering. This mainly involves processing the amplitude of the denoised signal, as shown in the Formula (11):


(11) $$S_{3}\left(i\right)=\mathrm{filter}\;\left(S_{2}\left(i\right),f_{\mathrm{cut}}\right)$$

Sub-process 4: Normalizing the sEMG signal obtained from process 3, as shown in the Formula (12):


(12) $$S_{4}\left(i\right)=\overset{N-1}{\underset{i=0}{\sum }}S_{3}\left(i\right)/MVC\left(1,S_{3}\left(i\right)\right)$$

Where $S_{4}\left(i\right)$ represents the signal amplitude, and MVC represents the maximum voluntary contraction strength of the muscle.

Sub-process 5: Smoothing the signal after normalization, as shown in the Formula (13):


(13) $$S_{5}\left(i\right)=\sqrt{\frac{1}{t_{i}-t_{i-1}}\overset{t_{i}}{\underset{t_{i-1}}{\int }}{\left[S_{4}\left(i\right)\right]}^{2}dt}$$

Where $S_{5}\left(i\right)$ represents the amplitude of the signal after processing, and $t_{i}-t_{i-1}$ represents the time difference value.

The sEMG signal obtained through the above five processing steps can directly reflect the changing characteristics of the sEMG signal, including the linear variation pattern of the sEMG signal amplitude.

After the original electromyographic (EMG) signals are collected, it is necessary to preprocess the EMG signals and extract features based on the processed signals. The purpose is to extract components of the EMG signals that can reflect the degree of fatigue. Different degrees of fatigue have their own characteristics, and the more representative the feature selection, the more accurate the pattern recognition. Based on the common time-domain and frequency-domain features of EMG signals and their clinical significance, this study selects four main features—mean power frequency (MPF), median frequency (MF), root mean square (RMS), and integrated electromyogram (IEMG)—to reflect the muscle’s fatigue state.

The time-domain features of muscle fatigue can be used to describe the amplitude changes in electromyographic (EMG) signals during the process of muscle fatigue. Calculating the integrated electromyogram (IEMG) and root mean square (RMS) can visually reflect this change. Let $y\left(t\right)$ represent the preprocessed original EMG signal, the calculation formulas are shown in Formula (14) and Formula (15):


(14) $$\mathrm{IEMG}=\overset{t+T}{\underset{t}{\int }}\left|y\left(t\right)\right|dt$$

(15) $$RMS={\left(\frac{1}{T}\overset{t+T}{\underset{t}{\int }}y^{2}\left(t\right)dt\right)}^{2}$$

The frequency-domain features of electromyographic (EMG) signals are obtained by transforming their time-domain signals into frequency-domain signals using Fourier transform, and then analyzing the signal’s power spectrum or frequency spectrum. The selected features in this study are median frequency (MF) and mean power frequency (MPF). Let $P\left(f\right)$ represent the power spectral density and $d\left(f\right)$ represent the signal’s frequency resolution, the calculation formulas are as shown in Formula (16) and Formula (17):


(16) $$\overset{MF}{\underset{f1}{\int }}PS\left(f\right)df=\overset{f2}{\underset{MF}{\int }}P\left(f\right)Sdf=P\left(f\right)Sdf=\frac{1}{2}\overset{f2}{\underset{f1}{\int }}PS\left(f\right)df$$

(17) $$MPF=\frac{\int_{f1}^{f2}fPS\left(f\right)df}{\int_{f1}^{f2}PS\left(f\right)df}$$

The similarity between the two lies in that the types of sEMG features covered are all included in the four features listed in this paper. The difference lies in the fact that general sports movements have larger amplitude and intensity but are relatively singular. This results in sports-related sEMG features showing large numerical values but being singular in type, usually consisting of 1–2 of the four features. However, during the driving operation of a hexapod robot driver, although the amplitude of movements is not large, the driver’s movements are more diverse and of longer duration, generally encompassing all four listed features. It is necessary to comprehensively analyze all four features to determine the driver’s fatigue state.

In order to establish and utilize a neural network model for real-time assessment of driver upper limb fatigue, this study recorded the electromyographic (EMG) signal data of several hexapod robot drivers while operating the hexapod robot, along with their subjective perception data of upper limb fatigue. A BP neural network was used to correlate the EMG feature data with the upper limb fatigue data. The subjective perception data of upper limb fatigue come from the drivers’ self-rated mental fatigue scores, where higher scores indicate higher levels of current mental fatigue. The feature data of the EMG signals, which include various information such as EMG integral value, median frequency, root mean square value, change when the muscles are fatigued, were used as inputs. Participants’ comprehensive fatigue values were provided as outputs for model training. The training model includes input layer, output layer, and intermediate layers. A total of 500 sets of data were collected from different participants, with 400 sets chosen for training and 100 sets for testing. The training set includes EMG feature data and drivers’ subjective fatigue levels. An intermediate layer was set up, and during the training process, the connection weights and thresholds of each layer were calculated to obtain the network model for predicting driver arm fatigue (DAF).

Since drivers may still persist in operating the vehicle with mental strength when experiencing muscular fatigue, sEMG features may not reflect the driver’s mental fatigue at this time. Considering that the driver’s mental fatigue can be reflected by operational error rate and trajectory deviation, this paper utilizes the Driver Manipulation Error Rate (DMER) and Trajectory Offset Rate (TOR) to assist sEMG features in determining the driver’s fatigue state together.

Specifically, the Driver Manipulation Error Rate (DMER) is used to describe the rate of inappropriate manipulation by the driver when issuing control commands to the hexapod robot. Let $I_{0}^{\left(k\right)}$ represent the set of instructions given by the driver in a non-fatigued driving state for a particular terrain, and let $I_{i}^{\left(k\right)}$ represent the control instructions given in different fatigue states on the same terrain, where *k* = 1, 2… *N* and *i* = 1, 2… *n*. *N* represents the number of instruction sets, and n represents the number of different fatigue states. The MER can be expressed as Formula (18):


(18) $$MER=\frac{\overset{n}{\underset{i=1}{\sum }}\overset{N}{\underset{k=1}{\sum }}\frac{I_{i}^{k}-I_{0}^{k}}{I_{0}^{k}}}{N\cdot n}$$

Specifically, the Trajectory Offset (TO) can be described as the deviation of the actual path traveled by the hexapod robot from the average trajectory while being driven by the driver. Assuming the sampling interval for the distance traveled by the hexapod robot is T, the average speed of the hexapod robot within this interval is v, and the number of samples is N, with the actual position traveled denoted as S, the trajectory offset TO can be expressed as Formula (19):


(19) $$TO=\overset{N}{\underset{j=1}{\sum }}vT\left(S_{j}-\frac{\overset{N}{\underset{i=1}{\sum }}S_{i}vT}{N}\right)$$

## 4 Experiment

**Figure 9:**
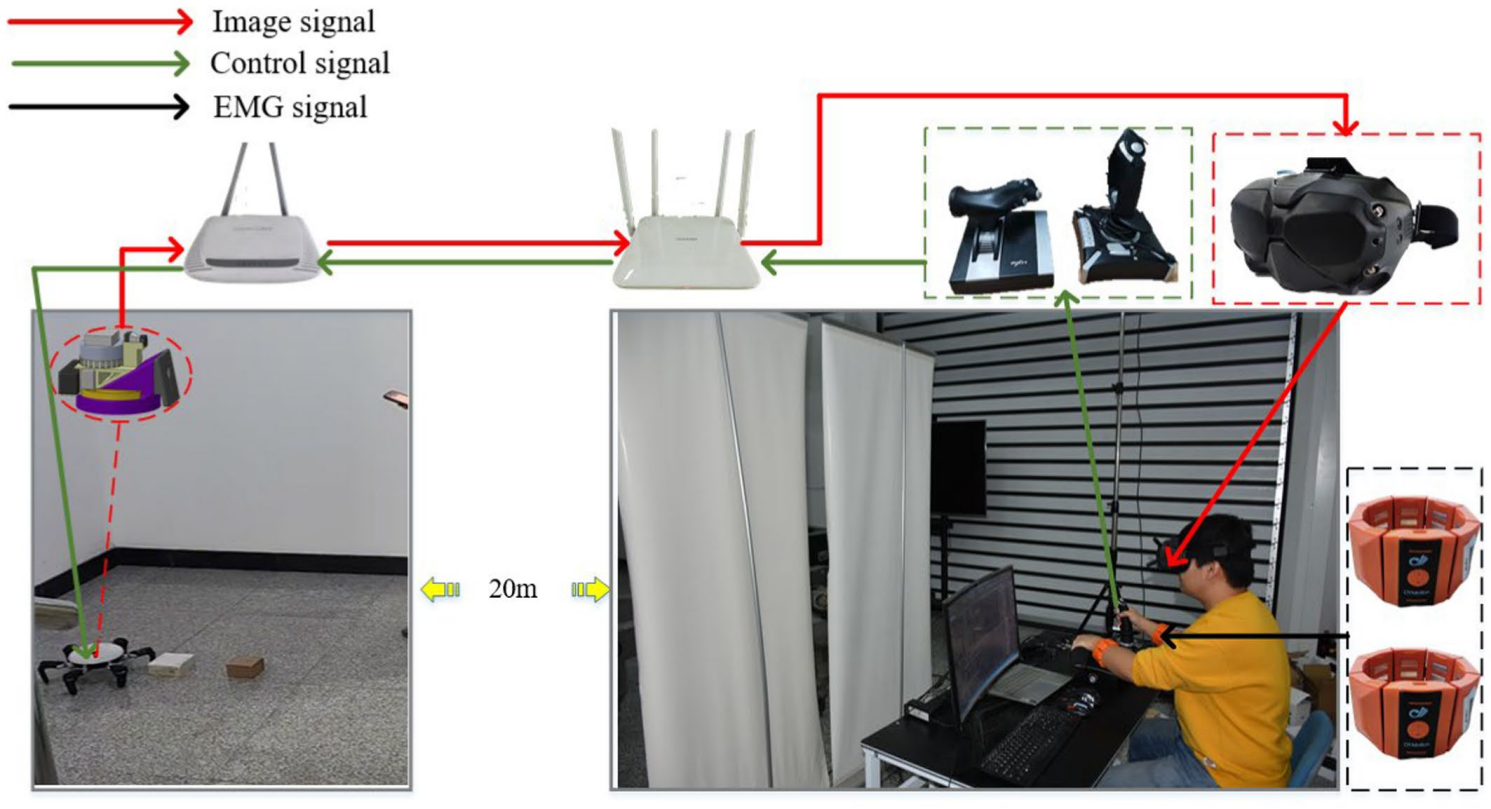
The remote human-machine collaborative driving control system.

In order to enable drivers to remotely control hexapod robots in a human-machine collaborative manner and validate the effectiveness of the proposed method, this study has built a remote human-machine collaborative driving control system based on wearable VR glasses, EMG armbands, and other human-machine interaction devices. Using wireless network signals, drivers can control the robot from an operating platform 20 meters away. Specifically, considering that the perception and feedback loops between humans and machines constrain the efficiency of human-machine collaborative decision-making, to effectively enhance the depth of human-machine integration, this study processes robot visual camera data and transmits it to virtual reality devices, allowing drivers to experience immersive driving from a first-person perspective. Additionally, drivers wear EMG armbands on both arms to monitor upper limb fatigue in real time. The system has successfully integrated various control hardware such as multifunctional joysticks, throttle levers, touchscreens, etc., greatly enhancing the driver’s sense of presence during remote driving control and enabling better collaborative decision-making tasks with the robot. The remote human-machine collaborative driving control system described above is shown in the Figure 9.

**Figure 10:**
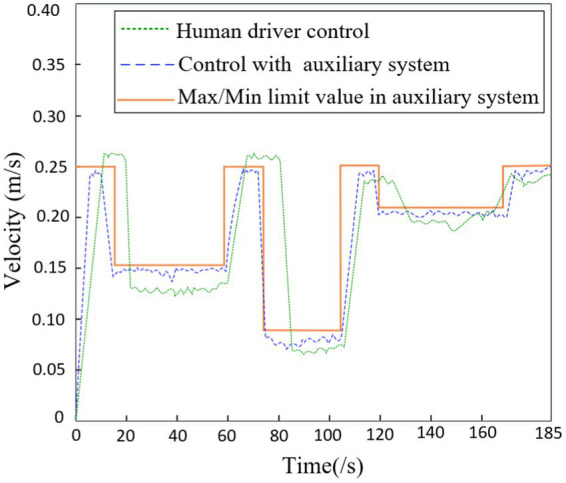
The velocity of robot in terrain.

Based on the remote human-machine collaborative driving control system described above, this study conducted physical experiments on integrated terrains with obstacles, gullies, and slopes. As shown in Figure 10, the speed of the robot’s center of mass when driven alone through integrated terrains is recorded (green dashed line). In addition, with the assistance of the auxiliary system proposed in this study, the driver navigates through integrated terrains and records the speed of the robot’s center of mass (blue dashed line). The auxiliary system can generate a feasible set of instructions in real-time during the robot’s travel based on decision-making target values, and, after filtering through constraint conditions, provide real-time recommended human-machine instructions to improve the efficiency of driver instruction issuance. The red solid line in the figure represents the maximum limit value of the center of mass speed prompted by the auxiliary system. It can be observed that from 0 to 18 s, the robot walks on flat terrain, during which the maximum prompted speed limit for the center of mass by the auxiliary system is 0.25 m/s. From 18–60s, the robot moves through obstacle terrain, during which the prompted maximum speed limit for the center of mass by the auxiliary system decreases to 0.15 m/s. From 60 to 78 s, the robot once again walks on flat terrain, during which the prompted maximum speed limit for the center of mass by the auxiliary system returns to 0.25 m/s. From 78 to 106 s, the robot walks through gully terrain, during which the prompted maximum speed limit for the center of mass by the auxiliary system decreases to 0.09 m/s. From 106 to 122 s, the robot walks on flat terrain, during which the prompted maximum speed limit for the center of mass by the auxiliary system is 0.25 m/s. From 122 to 170 s, the robot walks on uphill terrain, during which the prompted maximum speed limit for the center of mass by the auxiliary system decreases to 0.09 m/s. From 170 to 185 s, the robot walks on flat terrain, during which the prompted maximum speed limit for the center of mass by the auxiliary system returns to 0.25 m/s. In summary, when the driver navigates alone through integrated terrains, there is a slight lag in speed control during transitional phases of terrain changes. However, after adopting the auxiliary system for maximum speed prompts, the driver can promptly adjust the speed before the terrain changes and, knowing the maximum speed limit, can also raise the speed in real-time in a reasonable manner, ensuring travel safety and efficiency.

**Figure 11:**
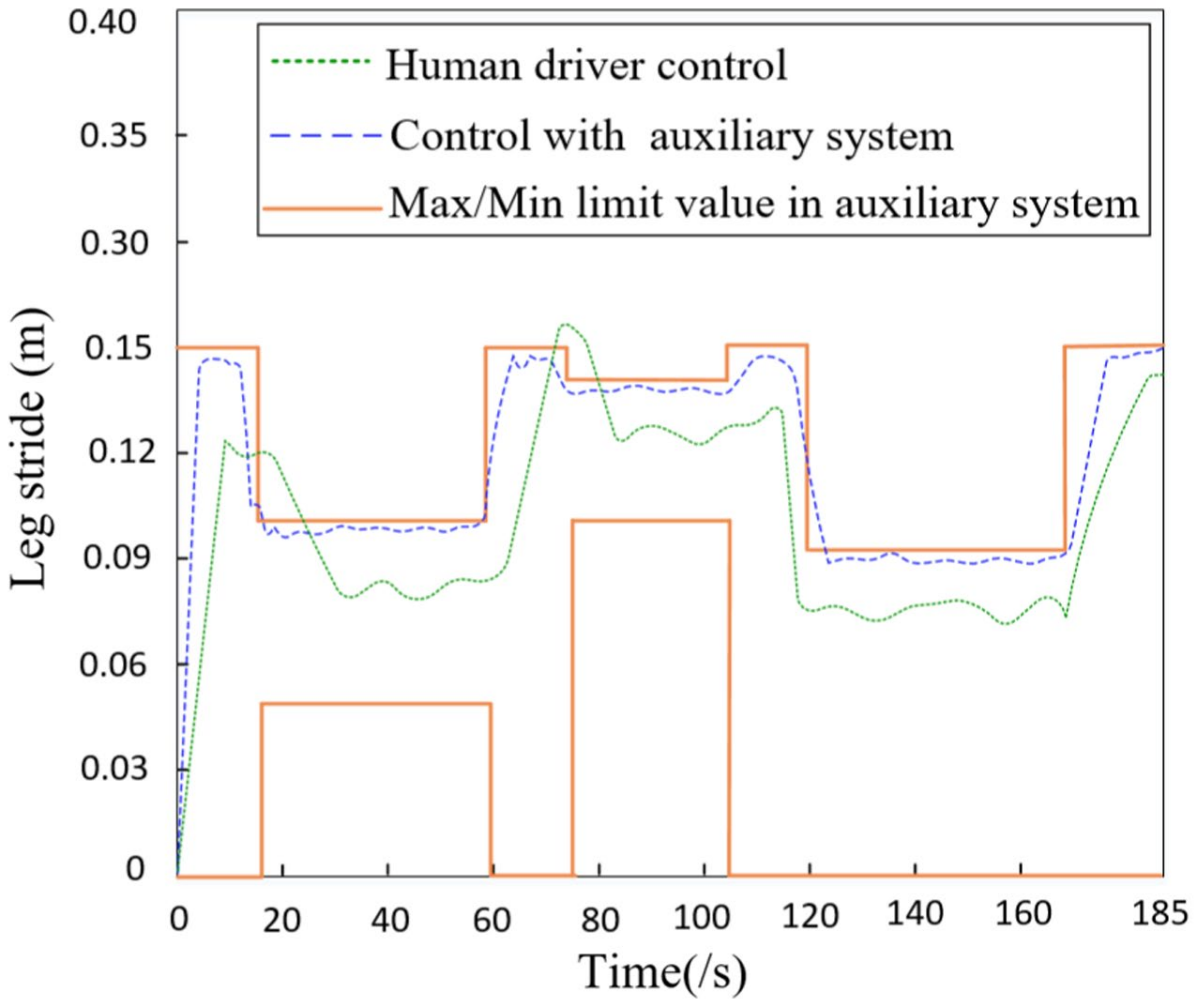
The leg stride of robot in terrain.

As shown in Figure 11, the robot’s stride when driven alone through integrated terrains is recorded (green dashed line). Additionally, with the assistance of the auxiliary system proposed in this study, the driver navigates through integrated terrains and records the robot’s stride (blue dashed line). The red solid line represents the maximum and minimum limit values prompted by the auxiliary system. It can be observed that from 18–60s, the robot walks on obstacle terrain, during which the maximum stride limit prompted by the auxiliary system is 0.1 m and the minimum limit is 0.5 m; from 78 to 106 s, the robot walks on gully terrain, during which the maximum stride limit prompted by the auxiliary system is 0.14 m and the minimum limit is 0.1 m; from 122 to 170 s, the robot walks on uphill terrain, during which the maximum stride limit prompted by the auxiliary system is 0.09 m. In summary, when the driver navigates alone through integrated terrains, there is a slight lag in controlling the robot’s stride during transitional phases of terrain changes. However, after using the auxiliary system for maximum stride prompts, the driver can promptly adjust the stride before the terrain changes and, knowing the real-time limits of the stride, can increase it in a reasonable manner in real time, further ensuring travel safety and efficiency.

**Figure 12:**
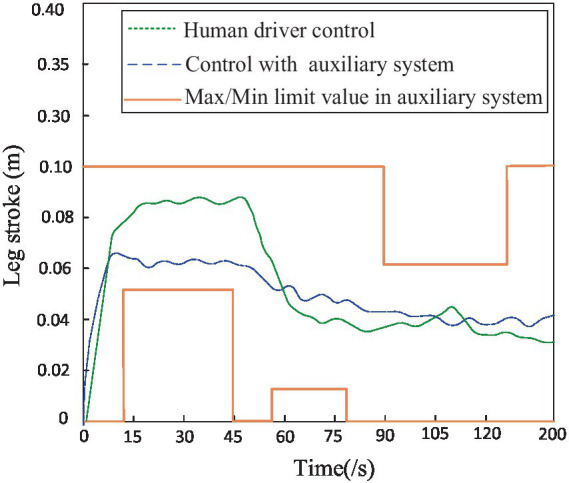
The leg stroke of robot in terrain.

As shown in Figure 12, the robot’s step height when driven alone through integrated terrains is recorded (green dotted line). Additionally, with the assistance of the auxiliary system proposed in this study, the driver navigates through integrated terrains and records the robot’s step height (blue dashed line). The red solid line represents the maximum and minimum limit values prompted by the auxiliary system. It can be observed that from 18 to 60 s, the robot walks on obstacle terrain, during which the minimum step height limit prompted by the auxiliary system is 0.05 m; from 78 to 106 s, the robot walks on gully terrain, during which the minimum step height limit prompted by the auxiliary system is 0.01 m; from 122 to 170 s, the robot walks on uphill terrain, during which the maximum step height limit prompted by the auxiliary system is 0.06 m. In conclusion, compared to driving alone through integrated terrains, using the auxiliary system for maximum step height prompts allows for real-time reasonable reduction in step height based on the known real-time limit values, further ensuring travel safety and efficiency.

**Figure 13:**
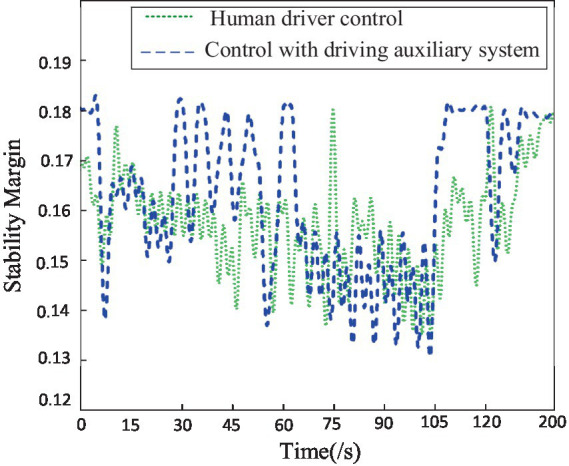
The stability margin of robot in terrain.

**Figure 14:**
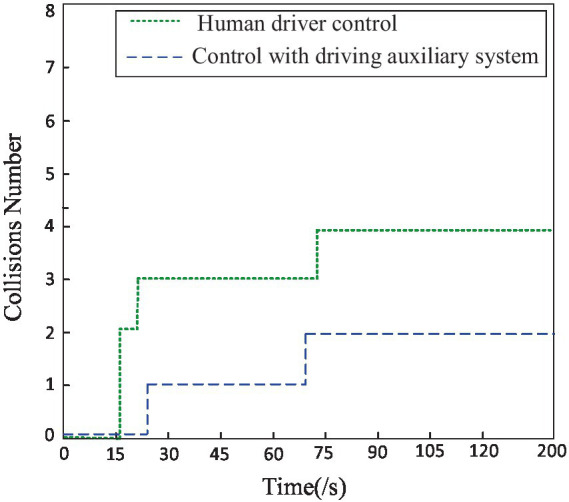
The collision numbers of robot in terrain.

To further compare the impact of driving alone versus using a driving assistance system on the performance of the hexapod robot, this study utilized two driving evaluation indicators: static stability margin and collision coefficient. The stability was quantitatively evaluated during the robot’s travel process using established static stability margin evaluation standards, while the collision count was determined and defined by detecting the pausing of swinging legs. As shown in Figure 13, it can be observed that when the driver utilizes the driving assistance system to remotely control the robot, the average stability is higher than the average stability when driving alone. This is particularly evident when traversing obstacle and uphill terrains, where the robot’s stability is significantly higher than when driven alone, as depicted in Figure 14. When the driver utilizes the driving assistance system for remote control, the collision count between the robot and the environment is noticeably lower than when driving alone, especially when traversing obstacle and gully terrains. Further analysis indicates that, compared to human driving alone, a hexapod driver with the assistance of auxiliary systems improves robot stability by 12.5% and reduces the number of collisions between the robot and the surrounding environment by 50%.

Based on the analysis of the experimental results, it can be seen that the assistance of auxiliary systems provides command combinations, shifting the driver’s task from making decisions to making choices, effectively reducing the driver’s decision-making burden. At the same time, by providing the extreme values of each command, not only can it enhance the safety of robot locomotion, but it also to some extent improves the robot’s moving speed and traffic efficiency in complex environments.

## 5 Conclusion

The most important achievement in this paper is the development of a novel neural human-robot command combination method for improving the hexapod robot’s walking performance and reducing the burden on drivers’ control. This article first proposes a mapping process that generates human-robot command combinations from decision target values, focusing the robot intelligence on assisting drivers by generating human-robot instruction combinations. In addition, this article quantifies robot motion geometric constraints and driver fatigue constraints. By using constraints to optimize and filter the feasible set of human-robot commands, a small number of human-machine command combinations are formed. A human-robot command assistance recommendation system is developed to provide real-time recommendations of human-robot command combinations to drivers. The results of the designed experimental platform demonstrate the validity of the human-robot command assistance recommendation system. In the future, considering the situation where both humans and machines have operational authority over the same command combination, we will continue to research human-robot command fusion based on the game theory.

## Data availability statement

The datasets presented in this article are not readily available because they involve the hexapod robot or a robot with the same configuration. Requests to access the datasets should be directed to cxl_920101@163.com.

## Author contributions

XC: Methodology, Writing – original draft. BY: Funding acquisition, Writing – review & editing. ZD: Software, Writing – review & editing.
